# Lifestyle as a predictor for colonic neoplasia in asymptomatic individuals

**DOI:** 10.1186/1471-230X-6-5

**Published:** 2006-01-13

**Authors:** Inger K Larsen, Tom Grotmol, Kari Almendingen, Geir Hoff

**Affiliations:** 1 The Cancer Registry of Norway, Institute of Population-based Cancer Research, Montebello, N-0310, Norway; 2 Rikshospitalet, University Hospital, Oslo, Norway

## Abstract

**Background:**

Lifestyle is a well-established risk factor for colorectal cancer (CRC) and is also found to be associated with occurrence of adenomas. In the present study we evaluated risk factors for both low-risk adenomas and advanced neoplasia in asymptomatic individuals using a single-paged questionnaire. Aiming to see if the questionnaire was a useful tool in picking up high-risk individuals.

**Methods:**

A cross-sectional study was carried out within a randomised controlled colorectal cancer screening trial (n = 6961). The population comprised men and women born between 1946 and 1950. Before screening in year 2001 they were asked to fill in a questionnaire about their present lifestyle. Cases were categorised according to the most severe findings at screening. Analyses were then conducted to find risk factors associated with the presence of either low-risk adenomas or advanced neoplasia.

**Results:**

The response rate among attendees was 97% (3998/4111). Among these, 3447 (86%) had no neoplasia, 443 (11%) had low-risk adenomas, and 108 (3%) had advanced neoplasia. Low-risk adenomas were significantly associated with current smoking, and obesity. Participants with advanced neoplasia had a two-fold increased risk of not adhering to any of the selected lifestyle recommendations compared to controls. However, current smoking was the only variable that reached statistical significance in the multivariate analysis for these lesions. A dose-response relationship to the consumption of cigarettes per day was shown, where OR was 2.04 (CI 1.07–3.89) for the lowest consumption category.

**Conclusion:**

The present findings indicate that a short questionnaire may be adequate in picking up the most consistent associations between lifestyle risk factors and colorectal neoplasia. Smoking and BMI were found to be the most significant risk factors for neoplasia, but adhering to recommendations on diet, and physical activity seems also to be of importance.

## Background

Colorectal cancer (CRC) is one of the commonest cancer diseases in developed countries [1]. It develops through multiple molecular steps, usually through a stage of adenomatous polyp [2], and the risk of both CRC [3-5] and adenomas [6-11] is shown to be associated with lifestyle factors such as smoking, dietary habits, physical inactivity and overweight. Three randomised trials have shown that CRC screening intervention groups have reduced risk of CRC mortality [12,13] and incidence [14]. In a large-scale randomised screening trial in Norway, 21,000 individuals were drawn by randomisation from the population registry and invited to have a once-only Flexible Sigmoidoscopy (FS) examination (The Norwegian Colorectal Cancer Prevention study – NORCCAP). The main objective was to investigate the effect of FS screening on CRC incidence and mortality. Design and baseline findings are described in detail elsewhere [15,16]. A major challenge for the NORCCAP trial was to include a number of relevant sub-studies without jeopardizing the attendance rate. Comprehensive pre-screening questionnaires could be a serious threat to attendance and were therefore not accepted. One of the studies requiring a questionnaire on lifestyle, was a project looking into possible undesirable effects on lifestyle caused by screening programmes [17]. For this purpose we designed a simple, single-paged questionnaire, and the object of this study was to evaluate risk factors for neoplasia in asymptomatic individuals using this questionnaire.

## Methods

### Design and study population

The NORCCAP-trial is a large randomised trial in which men and women (1:1), aged 50–64, living in Telemark (mixed rural and urban population) or Oslo (urban population) (1:1) were randomly drawn from the national population registry to be invited to have a colorectal cancer screening examination. The screening group was randomised to two screening arms (1:1) of either a once-only FS examination or a combination of once-only FS and a Fecal Occult Blood Test (FOBT). Sampling for FOBT was done at home no earlier than 10 days before attendance and collected from three subsequent stool samples. Participants with positive FS or FOBT (n = 2639) were given an appointment for colonoscopy, and 2524 (19%) persons attended for a colonoscopy work-up. Sometimes the colonoscopy was repeated due to inadequate initial examination or incomplete polypectomy. Findings from baseline colonoscopy and supplementary work-up examinations are included in the present analyses. The trial started January 1999, and during a three-year period, 20,780 subjects were invited to screening of whom 777 were excluded according to exclusion criteria. Altogether 12,960 attended (65%). Forty-one (0.3%) cases were diagnosed with CRC. Five hundred and forty-five (4.2%) subjects had high-risk adenomas, whereas 2208 (17%) cases were diagnosed with any adenoma. Significantly higher attendance rates were observed in the arm with FS screening only (67%), compared to the combined screening arm (63%), among women (66%) compared to men (64%), in Telemark (71%) compared to Oslo (58%), and in the oldest age group (60–64 year) (67%) compared to the youngest (50–54 year) (62%) [16].

**Figure 1:**
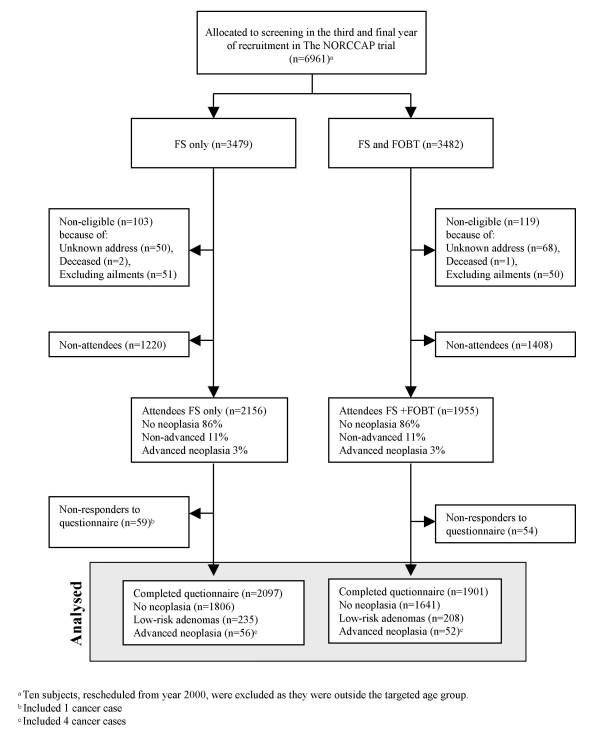
Flow chart of participation and response to the questionnaire on lifestyle in the screening group.

The target population for the present cross-sectional study was men and women, born between 1946 and 1950 (aged 50–55), and invited to the third and final year of recruitment (January–December 2001) in the NORCCAP study (n = 6961) (Figure 1). Participants, who were excluded from the screening trial according to the criteria below, were also excluded from the present sub-study.

### Exclusion criteria

The following ones were excluded: Patients with previous open colorectal surgery (resections, enterostomies); individuals in need of long-lasting attention and nursing care (somatic or psychosocial reasons, mental retardation); on-going cytotoxic or radiotherapy for malignant disease; severe chronic cardiopulmonary disease (NYHA III-IV); patients on life-long anticoagulant therapy (warfarin); a coronary episode requiring hospital admission during the last 3 months; a cerebrovascular accident during the last 3 months; resident abroad or postal return of unopened mail marked 'address unknown' or 'dead'.

### Examination procedures

The examination procedures are described in detail elsewhere [15]. Briefly, visible polypous lesions were biopsied or removed by polypectomy and sent for histopathological diagnosis. A positive FS, defined as any polyp ≥10 mm or a finding of any bioptically verified neoplasia, irrespective of its size, qualified for colonoscopy. In the combined screening arm (FS+FOBT), a positive FOBT also qualified for colonoscopy. Patients were classified according to the histologically most advanced lesion. An advanced neoplasia was defined as cancer or an adenoma measuring ≥ 10 mm in diameter and/or with villous components (villous or tubulovillous) and/or showing severe dysplasia.

### Questionnaire

Questions and reply options are summarised in the Additional file 1. Upon attendance at the screening centres, and before being exposed to bowel preparation and interviews, participants were asked to fill in a self-reporting single-paged questionnaire about their present lifestyle. A working group, consisting of physicians, statisticians and nutrition researchers, designed the questionnaire. Several of the questions were chosen from questionnaires validated and extensively used in national health surveys [18]. The questionnaire was designed for detection of temporal changes in lifestyle factors known to be associated with an increased risk of cardiovascular disease (best documented and most prevalent cause of lifestyle related deaths), and was estimated to take approximately 10 minutes to be completed. This included risk factors such as body mass index (BMI (kg/m^2^)), smoking habits, physical activity and some dietary variables, all of which are associated with colorectal cancer and adenomas.

BMI was calculated from self-reported body weight and height, and categorized as normal (BMI<25 kg/m^2 ^(which included 40 participants with BMI≤18.5 kg/m^2^)), overweight (BMI 25.0–29.9 kg/m^2^), or obese (BMI≥30 kg/m^2^).

Smoking behaviour was categorised into six groups: 'never smokers'; 'past smokers'; 'occasional smokers'; 'daily smokers consuming 1–10 cigarettes per day'; '11–20 cigarettes per day' and 'more than 20 cigarettes per day'. In the group of occasional smokers, 88 individuals also specified a certain level of daily cigarette consumption. This, and similar inconsistencies, forced us to give a subjective encoding on some answers (see Additional file 1). No analyses were performed on pipe and cigar smokers alone as these were few (n = 99), and most of them also stated to be past (n = 4), occasional (n = 32) or current cigarette smokers (n = 54).

All analyses concerning physical activity were carried out on a variable expressing a total score for exercise (min 2 – max 12). This score was calculated from reported frequencies of performing moderate (exercise without sweating) and vigorous (exercise with sweating) physical activity (see Additional file 1 for details).

Questions on foods included items both from a traditional Norwegian diet and dietary recommendations [19]. The mean calculations are based on data categorised as 1 = [Never]; 2 = [1–3 times/month]; 3 = [1–3 times/week]; 4 = [4–6 times/week]; 5 = [1–2 times/day]; 6 = [>3 times per day]. Additionally, vegetable, fruit and berry consumptions were merged into a new variable expressing the mean servings of greens per day (specified in the Additional file 1).

In the regression analyses, each item was categorised into three frequency groups, except for vegetable, fruit and berry consumptions, which were grouped into four frequency categories. The consumption of boiled potatoes, which is demonstrated to be 20–45% higher in Norway than in other European countries [20], was analysed separately from other vegetables.

Five lifestyle variables; BMI, smoking, physical activity with and without sweating, and consumption of greens were chosen to assess the general lifestyle. The variables were classified as adherence to recommendations if; BMI <25 kg/m^2^, physical activity with and without sweating ≥ 3 times per week, current non-smoking (which included past smokers) and servings of greens ≥ 5 times per day.

### Analysis

Screenees were categorised into three groups: 'controls with no adenoma', and participants with either 'low-risk' or 'advanced neoplasia'. Mean of each lifestyle variable was estimated for the three categories, and a test for linearity was performed to measure the goodness of fit across the categories. In these analyses, the groups were stratified by gender.

Univariate logistic regression analysis was carried out to estimate relative risk, expressed as odds ratios (ORs). Additionally, univariate analyses were adjusted for gender, and these analyses are only referred to when major differences in the significance level were observed. Multiple logistic regression analyses were performed with an adjustment for age, gender, BMI, smoking category, total score for exercise and the consumption of vegetables, fruit and berry, boiled potatoes, fish and poultry or other meat.

A multivariate logistic regression sub-analysis was carried out for screenees that stated to have sustained their dietary habits for at least one year. The degree of association between the lifestyle variables was expressed by the Spearman rank correlation coefficient (r_s_). All p-values were two-tailed, and p < 0.05 was considered significant. We applied the statistical software SPSS 11.5 (SPSS, Chicago, IL., USA).

### Ethics

The Regional Research Ethics Committee and the Data Inspectorate approved the study protocol. Written informed consent was obtained from all participants.

## Results

### Attendance and population description

A flow-chart of the trial is given in Figure 1. Two hundred and thirty-two participants were defined as non-eligible because of unknown change of address (n = 118), deceased (n = 3), excluded according to the exclusion criteria (n = 101) or late entries from older age groups who should have been screened the previous year (n = 10). The attendance rate among eligible subjects (n = 6739) in the present study was 61%. Non-attendance was 36% in the FS only arm, and 43% in the combined FS and FOBT arm (Details are given in Figure 1). Almost all of the attendees (97% (3998/4111)) replied to the questionnaire on lifestyle. Among these, a total of 3447 (86%) had no neoplasia, 443 (11%) had low-risk adenomas, and 108 (3%) had advanced neoplasia (including 8 cases of CRC). One case of CRC was detected among the 3% of participants that did not respond to the lifestyle questionnaire. The demographic characteristics of the study population are shown in Table 1.

### Lifestyle factors associated with low-risk adenomas

In the univariate analyses, current smoking, BMI, consumption of potatoes and meat (other than poultry) at least four times per week, were all positively associated with the presence of low-risk adenomas. A negative association was found for the highest quartile of vegetables and fruit consumption (Table 2). After adjustment for gender, the associations with BMI and servings of greens were no longer significant.

In the multivariate analysis, low-risk adenomas were again positively associated with the highest category of BMI compared to the lowest (OR 1.57, CI 1.13–2.18) (Table 3). Former, occasional or current smoking were also associated with an increased risk compared to never smokers, but the association was only significant for current smokers consuming 11–20 cigarettes per day (OR 1.76, CI 1.29–2.40).

A change in dietary habits during the last year was stated by 19%. Of these, 75% had changed to a healthier diet and 2% to an unhealthier one, while 23% could not be classified as healthy or unhealthy. Twenty-seven percent of those who had changed to a healthier diet pointed out that this was caused by a recent diagnosis of diabetes mellitus, cardiovascular disease, or as an attempt to reduce weight. Our findings on low-risk adenomas were not changed by restricting the multivariate analysis to screenees stating no dietary changes during the last year (data not shown).

### Lifestyle factors associated with advanced neoplasia

In univariate analysis, current smoking had the strongest association with the presence of advanced lesions. Screenees in the lowest category of current daily cigarette consumption (1–10 cigarettes per day) had a two-fold increased risk of advanced lesions compared to never smokers (Table 2). A clear dose-response relationship was found as screenees with a daily consumption of more than 20 cigarettes per day had a four-fold increased risk of advanced lesions compared to never smokers. No significant association was found for former smokers.

For women, there was a significant trend (p = 0.03) of increased mean BMI across the three categorical groups: no neoplasia (25.2 kg/m^2^), low-risk adenomas (25.5 kg/m^2^) and advanced neoplasia (26.7 kg/m^2^) (Table 1). No significant trend towards increased BMI across the groups was observed among men. Participants with advanced neoplasia (women and men combined) had the highest proportion of obesity (15.7% compared to 11.7% in the control group (Table 2)). For men and women combined, the association between BMI and advanced neoplasia was not significant neither in the univariate nor the multivariate analyses (Tables 2 and 3).

In the univariate model, a negative association was found for screenees in the highest quartile of total score for physical exercise compared to the lowest (OR 0.56, CI 0.34–0.92) (Table 2). Fruit and vegetable consumption was also found to be negatively associated with advanced neoplasia, as screenees in quartiles 3 and 4 had more than 50% reduced risk of advanced lesions compared to screenees in the lowest category of consumption. For men only, these findings were also demonstrated by the test for linearity across the three categories (Table 1). In the multivariate model, however, no lifestyle variable was found to be negatively associated with advanced neoplasia, and current smoking remained as the only variable associated with the presence of these lesions (Table 3).

Notably, when the multivariate analysis on advanced neoplasia was carried out exclusively on screenees with no change in dietary habits during the last year, a significantly increased risk of advanced neoplasia was only found for smokers consuming 10–20 cigarettes per day, and a decreased risk was found for the highest category of total exercise score compared to the lowest (OR 0.48, 0.25–0.92, data not shown in table). No significant association was found between advanced neoplasia and the consumption of vegetables and fruit.

### Correlation between lifestyle variables

**Figure 2:**
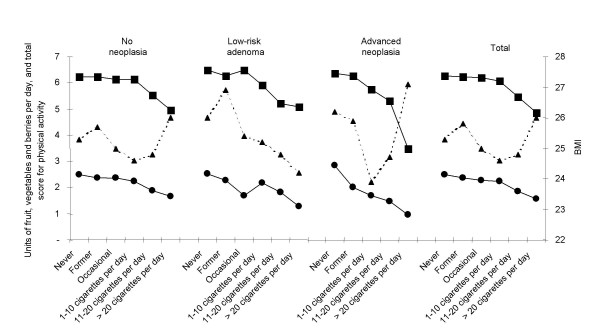
The score of greens consumption (mean), physical activity (mean) and BMI (median) according to neoplasia status and smoking behaviour.  Servings of fruit, vegetables and berries per day.  Total score of physical activity.  BMI.

Figure 2 shows some essential lifestyle variables according to neoplasia status and smoking behaviour. Several risk factors were strongly correlated to each other. In general, never and former smokers had the highest total score for physical activity and intake of vegetables, fruit and berries. A negative correlation was observed between an increased unfavourable smoking behaviour and the total score for exercise (r_s _= -0.14, p < 0.001). A negative correlation was also found between smoking and the consumption of fruit (r_s _= -0.20, p < 0.001), uncooked vegetables (r_s _= -0.13, p < 0.001), and BMI (r_s _= -0.08, p < 0.001). No significant correlation was found between smoking and consumption of boiled vegetables.

**Figure 3:**
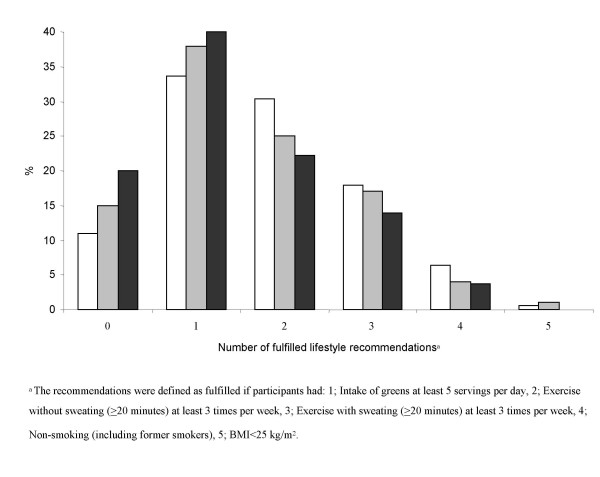
The proportion of participants, classified according to their neoplasia status, who fulfilled the lifestyle recommendations^a^.  No neoplasia.  Low-risk adenoma.  Advanced neoplasia.

The mean numbers of recommendations adhered to were 1.8, 1.6, and 1.4, among controls and participants with either low-risk or advanced neoplasia, respectively (test of linearity, p < 0.001). Very few followed the whole range of recommendations (n = 22 (0.6%)), particularly among those with advanced neoplasia. Actually, 20% of participants with advanced neoplasia did not adhere to any of the recommendations (22/108), and this was a significantly higher rate compared to controls (OR 2.02, CI 1.25–3.26; data not shown), (Figure 3).

## Discussion

In the present study, BMI along with current smoking were the only lifestyle variables positively associated with the presence of low-risk adenomas, and current smoking was found to be the only significant risk factor for advanced lesions. In the univariate analyses, our simplified food frequency questionnaire did demonstrate a significant association between low intake of vegetables, fruit and berries and a finding of low-risk or advanced neoplasia, but was not withstanding the critical test of multivariate analysis.

Ninety-seven percent of all eligible attendees were willing to fill in the questionnaire. For practical purposes, this eliminated any self-selection bias that might have been created if the questionnaire compliance had been low. This left us with a possible self-selection bias created by a choice to attend for screening examination or not. An association has been proposed between attendance rate and the prevalence of colorectal neoplasia among attendees [21], where those that are conscious of a familial risk may be the first to attend for screening, and the most reluctant ones may be those with the highest lifestyle-risk of CRC. Eligible attendees in the present sub-study comprised nearly 60% of the population sample invited to screening. Recently we have reported that attendees in this study were more physically active, and showed more adherence to general dietary recommendations compared to controls who were not invited for screening, thus supporting the hypothesis that individuals with low-risk lifestyle are more likely to attend [22]. On the other hand, they were more likely to be moderate smokers compared to controls, indication that some high-risk factors may also be prevalent in attendees.

Although bias was limited in the present study, misclassification by using FS rather than "gold standard" colonoscopy as a screening modality has inevitably misclassified some individuals with proximal neoplasia as adenoma-free. In the current trial, any adenoma detected at FS examination, irrespective of size, was offered a full colonoscopy. Results from colonoscopy screening studies, suggest that FS, with the same threshold for colonoscopy as in the present study, allows detection of 70–80% of all patients with advanced neoplasia [23,24]. If 551 individuals in our study (total number with low-risk and advanced neoplasia) represent 70% of attendees with neoplasia, then we may estimate 236 of our 3447 neoplasia-free screenees (7%) to be misclassified. It is more likely that the misclassification is lower. This is because there is an age-dependent proximal shift in the distribution of colorectal neoplasia, verified in the NORCCAP trial, reporting that age ≥60 years was significantly associated with the presence of proximal advanced neoplasia [25]. A colonoscopy screening study where the patient's mean age was 62.9 years found that 3.7 percent of the patients that had no adenomas in the rectum or sigmoid colon, did have advanced proximal lesions [23]. This argues for lower than 7% misclassification in our study, which probably is not likely to have substantially increased the risk of a type 2 statistical error.

Westernised diet is thought to be a major determinant of risk of colon cancer [26]. We could not find any dietary items to be associated with an increased risk of advanced neoplasia, while consumption of meat (other that poultry) or potatoes at least four times per week were associated with an increased risk of low-risk adenomas. In a screening study including more than 300 participants with advanced neoplasia, a highly significant association was found for participants consuming beef, pork or lamb more than five times per week compared to those who had not consumed these products [8]. In our study, regardless of neoplasia status, 68–69% had meat (other than poultry) 1–3 times per week. The relatively small number of cases with advanced neoplasia, together with the narrow distribution in meat consumption, may have limited the possibility to identify meat consumption as a risk factor for advanced lesions in this study. The negative association between consumption of fruit, berries and vegetables and the presence of neoplasia found in the univariate analyses was not maintained in the multivariate analyses. The association between CRC and fruit and vegetables consumption has not been consistently proven in other studies, as case-control studies have suggested a protective effect, while large prospective studies have not [27,28]. No substantial effect of dairy products or egg consumption was found on the development of colorectal polyps. Supporting this, a recently published systematic review found neither an association for dairy product nor egg consumption with the development of colorectal polyps [29]. Vitamin D has been observed to significantly reduce the risk of advanced neoplasia [8]. Fatty fish is a main source of dietary vitamin D, and was included in the present study as an indicator of vitamin D intake. Although we could not find a preventive effect of fatty fish consumption on the prevalence of neoplasia, the disagreement with previous findings may have been caused by our lack of information on vitamin D supplements.

To summarize regarding diet, our food frequency questionnaire is short and the level of precision may be low. It is not easy to decide whether or not this user-friendly questionnaire is suitable as a dietary measurement instrument, as results from comprehensive nutritional studies also have been inconclusive regarding the association between colon cancer and the intake of e.g. greens [28]. Probably, it is more likely that associations of diet and cancer risk are stronger when the dietary exposures are more heterogeneous [30].

The quality of BMI registration should not be influenced by the one-page design of our questionnaire. A number of studies have found a positive relationship between adenomas and BMI [31-33], and BMI has also been found to be associated with adenoma growth in a follow-up study [34]. In contrast, two studies presenting multivariate analyses with risk factors similar to ours, did not find any significant association between BMI and advanced neoplasia [8,9]. The majority of studies indicate that higher BMI increases the risk of CRC, and this association is also observed for adenomas. We found that mean BMI was highest among participants with advanced lesions, and they also had the highest proportion of participants categorised as obese. BMI was, however, only found to be an independent risk factor for low-risk adenomas. Notably, results including all participants during 3 years in the NORCCAP trial (aged 50–64)showed a positive association with BMI for low-risk (men and women) and advanced neoplasia. The association with advanced neoplasia was, however, found to be significant only for men [35]. In contrast, analyses in the present study, containing only the youngest age group in the NORCCAP trial, suggest a more pronounced positive association between BMI and neoplasia in women than men. These findings may reflect the women's menopausal status and estrogen level, since the effect of obesity is found to be less convincing for older women, and association between BMI and cancer are more consistently observed for men [32]. Furthermore, these findings may illustrate a limitation of this study, since the narrow age group restricts the study's generalization.

One interesting observation was that participants with advanced neoplasia twice as often did not adhere to any of the lifestyle recommendations including exercise and smoking habits, intake of greens, and BMI compared to controls. These results indicate a combined effect of adherence to the lifestyle recommendations, although the lifestyle variables, one-by-one, were non-significant predictors for neoplasia. However, present smoking was found to be the only significant risk factor for advanced neoplasia in the multivariate analysis. Smokers had a two- to three-fold increased risk for advanced neoplasia, which is in agreement with findings from other studies that have estimated the risk for colorectal adenomas [36]. Notably, we found that the association between smoking and low-risk adenomas was weaker than for advanced neoplasia. These findings support the concept of tobacco being an important risk factor also in the later stages of the adenoma-carcinoma sequence [5].

Finally, studies on gene-environment interactions have found that the impact of colorectal cancer risk factors, such as smoking, BMI, and vitamin D, is modified by specific genotypes [37-40]. As we expect that persons with a familial predisposition for CRC are more likely to participate in screening trials, it is reasonable to assume that the implication of genotypes and/or low-penetrance mutations have accounted for some of the cases that were not identified with risk factors included in our analyses.

## Conclusion

In conclusion, participants with advanced neoplasia seem to have a poorer lifestyle than controls, although, smoking was the only independent risk factor when adjusting for possible confounders for these lesions. Our results support the notion that tobacco is of importance also for the late steps of development of neoplastic lesions, as smoking was more strongly associated with advanced neoplasia than low risk adenomas. The present findings indicate that our short-version questionnaire may be adequate in picking up the most consistent associations between lifestyle risk factors and colorectal neoplasia when applied in a screening setting where the participants are unaware of presence or absence of neoplasia, and in a population sample of sufficient size.

## Competing interests

The author(s) declare that they have no competing interests.

## Authors' contributions

Inger Kristin Larsen is the corresponding author of the study. She took part in designing the study, performed the data analyses and drafted the manuscript. Tom Grotmol co-designed the study. He also supervised and co-drafted the manuscript.  Kari Almendingen co-designed the study, supervised the nutritional parts of the study, and gave critical comments to the manuscript. Geir Hoff had the idea and designed the study. He supervised and co-drafted the manuscript. All authors have read and approved the final version of the manuscript.

## Pre-publication history

The pre-publication history for this paper can be accessed here:



## Supplementary Material

Additional File 1 The additional file is a word file (name Appendix), in which all questions and reply options, used in this study, are summarised. Click here for file

## Figures and Tables

**Table 1:** Descriptive characteristics of the study population (n = 3998).

	Men (n = 1961)				Women (n = 2037)			
	No neoplasia (n = 1642)	Low-risk adenomas (n = 252)	Advanced neoplasia^a ^ (n = 67)	Test for linearity	No neoplasia (n = 1805)	Low risk adenomas (n = 191)	Advanced neoplasia^a ^ (n = 41)	Test for linearity

**Data below are given as mean (SD)**								
Mean age	53.06 (1.40)	53.06 (1.38)	53.36 (1.32)	ns	52.99 (1.41)	53.20 (1.44)	53.15 (1.33)	ns
Mean BMI (kg/m^2^)	26.18 (3.33)	26.38 (3.20)	26.36 (3.26)	ns	25.16 (4.16)	25.52 (4.27)	26.68 (8.45)	0.03
Median BMI	25.8 (3.33)	26.00 (3.20)	25.95 (3.26)	ns	24.50 (4.16)	25.10 (4.27)	24.00 (8.45)	0.03
Total score for exercise^b^	6.04 (2.02)	5.88 (2.07)	6.01 (2.04)	ns	6.12 (1.90)	6.21 (1.83)	5.25 (1.71)	ns
Servings of vegetables, fruit and berries per day	1.90 (1.41)	1.81 (1.28)	1.45 (1.00)	0.02	2.67 (1.86)	2.63 (1.79)	2.68 (2.12)	ns
Frequency of having^c^:								
Boiled potatoes	3.69 (0.83)	3.78 (0.80)	3.52 (0.68)	ns	3.65 (0.87)	3.75 (0.82)	3.58 (1.13)	ns
Poultry	2.26 (0.73)	2.23 (0.70)	2.13 (0.72)	ns	2.28 (0.75)	2.21 (0.65)	2.35 (0.92)	ns
Meat other than poultry	3.04 (0.69)	3.09 (0.70)	2.92 (0.59)	ns	2.94 (0.68)	3.05 (0.62)	2.97 (0.87)	ns
Fatty fish	2.68 (0.82)	2.57 (0.78)	2.61 (0.78)	ns	2.69 (0.83)	2.71 (0.82)	2.75 (0.93)	ns
Total consumption of milk (glasses/day)	1.53 (1.79)	1.55 (1.60)	1.54 (1.77)	ns	1.01 (1.46)	0.90 (1.42)	0.71 (0.98)	ns

**Data below are given in number and percentage within the group^d^**								
Smoking habits								
Never	534 (32.6%)	61 (24.2%)	11 (16.4%)		678 (37.7%)	62 (32.5%)	13 (31.7%)	
Former	522 (31.9%)	76 (30.2%)	17 (25.4%)		434 (24.1%)	40 (20.9%)	7 (17.1%)	
Occasional	82 (5.0%)	16 (6.3%)	1 (1.5%)		89 (4.9%)	9 (4.7%)	-	
1–10 cigarettes/day	222 (13.6%)	29 (11.5%)	12 (17.9%)		282 (15.7%)	42 (22.0%)	9 (22.0%)	
11–20 cigarettes/day	219 (13.4%)	57 (22.6%)	18 (26.9%)		277 (15.4%)	35 (18.3%)	12 (29.3%)	
>20 cigarettes/day	57 (3.5%)	13 (5.2%)	8 (11.9%)		40 (2.2%)	3 (1.6%)	-	
Percentage with chronic disease that limits level of physical exercise	290 (18.4%)	44 (18.3%)	15 (23.1%)		404 (23.5%)	49 (26.9%)	12 (32.4%)	
Percentage with changed dietary habits during the last year	291 (18.0%)	30 (12.0 %)	13 (20.0%)		385 (21.6%)	47 (24.7%)	10 (25.0%)	

^a^Including 4 cases of CRC

^b ^Minimum score = 2 and maximum score = 12

^c^The mean calculations are based on data categorised as 1 = [Never]; 2 = [1–3 times/month]; 3 = [1–3 times/week]; 4 = [4–6 times/week]; 5 = [1–2 times/day]; 6 = [≥3 times per day].

^d^The number of replies may not add up to the total, due to incomplete replies of the questionnaire.

**Table 2:** Observed numbers and crude odds ratios (ORs) of low-risk and advanced colonic neoplasia in association with various risk factors related to lifestyle (n = 3998)^a^.

	Controls^b ^(n = 3447)	Low-risk adenomas (n = 443)		**Advanced neoplasia **(n = 108)^c^	
Lifestyles variables^d^	n (%)	n (%)	OR [95% CI]	n (%)	OR [95% CI]

Gender					
Male	1642 (47.6%)	252 (56.9%)	Reference	67 (62.0%)	Reference
Female	1805 (52.4%)	191 (43.1%)	0.69 [0.57–0.84]	41 (38.0%)	0.56 [0.38–0.83]
					
BMI (kg/m^2^)					
BMI<25	1630 (47.6%)	187 (42.2%)	Reference	47 (43.5%)	Reference
BMI = 25–29.9	1392 (40.7%)	194 (43.8%)	1.22 [0.98–1.50]	44 (40.7%)	1.10 [0.72–1.66]
BMI ≥ 30	399 (11.7%)	62 (14.0%)	1.35 [1.00–1.84]	17 (15.7%)	1.48 [0.84–2.60]
			P_trend _= 0.02		P_trend _= 0.22
					
Smoking					
Never	1212 (35.3%)	123 (27.8%)	Reference	24 (22.2%)	Reference
Former	956 (27.8%)	116 (26.2%)	1.20 [0.92–1.56]	24 (22.2%)	1.27 [0.72–2.25]
Occasional	171 (5.0%)	25 (5.6%)	1.44 [0.91–2.28]	1 (0.9%)	0.30 [0.04–2.20]
1–10 cigarettes/day	504 (14.7%)	71 (16.0%)	1.39 [1.02–1.89]	21 (19.4%)	2.10 [1.16–3.81]
11–20 cigarettes/day	496 (14.4%)	92 (20.8%)	1.83 [1.37–2.44]	30 (27.8%)	3.05 [1.77–5.28]
>20 cigarettes/day	97 (2.8%)	16 (3.6%)	1.63 [0.93–2.85]	8 (7.4%)	4.17 [1.82–9.52]
			P_trend _< 0.001		P_trend _< 0.001
					
Total score for exercise with or without sweating					
Quartile 1 (score 2–4)	724 (22.1%)	99 (23.2%)	Reference	33 (31.1%)	Reference
Quartile 2 (score 5)	502 (15.4%)	69 (16.2%)	1.01 [0.72–1.40]	14 (13.2%)	0.61 [0.32–1.16]
Quartile 3 (score 6)	788 (24.1%)	93 (21.8%)	0.86 [0.64–1.17]	27 (25.5%)	0.75 [0.45–1.26]
Quartile 4 (score 7–12)	1256 (38.4%)	165 (38.7%)	0.96 [0.74–1.25]	32 (30.2%)	0.56 [0.34–0.92]
			P_trend _= 0.65		P_trend _= 0.04
					
Servings of vegetable, fruit and berries per day					
Quartile 1 (0–1.07 servings per day)	790 (24.1%)	108 (25.8%)	Reference	38 (36.9%)	Reference
Quartile 2 (1.08–1.71 servings per day)	784 (23.9%)	114 (27.2%)	1.06 [0.80–1.41]	27 (26.2%)	0.72 [0.43–1.18]
Quartile 3 (1.72–2.50 servings per day)	818 (24.9%)	109 (26.0%)	0.98 [0.73–1.29]	18 (17.5%)	0.46 [0.26–0.81]
Quartile 4 (At least 2.60 servings per day)	889 (27.1%)	88 (21.0%)	0.72 [0.54–0.98]	20 (19.4%)	0.47 [0.27–0.81]
			P_trend _= 0.03		P_trend _= 0.002
					
Consumption of boiled potatoes					
<1 time/week	234 (6.9%)	21 (4.8%)	Reference	9 (8.4%)	Reference
1–3 times/week	1082 (31.9%)	121 (27.7%)	1.25 [0.77–2.02]	33 (30.8%)	0.79 [0.37–1.68]
≥4 times/week	2075 (61.2%)	295 (67.5%)	1.58 [1.00–2.52]	65 (60.7%)	0.81 [0.40–1.66]
			P_trend _< 0.01		P_trend _= 0.75
					
Consumption of meat other than poultry					
<1 time/week	520 (15.4%)	55 (12.7%)	Reference	19 (18.3%)	Reference
1–3 times/week	2325 (69.0%)	295 (68.1%)	1.20 [0.87–1.62]	72 (69.2%)	0.85 [0.51–1.42]
≥4 times/week	523 (15.5%)	83 (19.2%)	1.50 [1.05–2.15]	13 (12.5%)	0.68 [0.33–1.39]
			P_trend _= 0.14		P_trend _= 0.76

^a^Subgroups may not total to 3998 because of missing values

^b ^No distal colorectal neoplasia diagnosed at FS

^c^Including 8 cases of screening detected CRC

^d^All variables are specified in the Additional file 1

**Table 3:** Adjusted odds ratio (OR) for having low-risk or advanced neoplasia compared to controls with no neoplasia diagnosed at FS depending on lifestyle characteristics among 3998 responders.

	Low-risk adenoma OR [95% CI]^a^		Advanced neoplasia OR [95% CI]^a^	
BMI (kg/m^2^)				
BMI<25		Reference		Reference
BMI = 25–29.9	1.11	[0.88–1.41]	0.94	[0.60–1.48]
BMI≥30	1.57	[1.13–2.18]	1.37	[0.72–2.58]
		P_trend _= 0.02		P_trend _= 0.61
				
Smoking				
Never		Reference		Reference
Former	1.06	[0.79–1.41]	1.30	[0.72–2.37]
Occasional	1.29	[0.78–2.15]	0.33	[0.04–2.45]
1–10 cigarettes/day	1.30	[0.93–1.83]	2.04	[1.07–3.89]
11–20 cigarettes/day	1.76	[1.29–2.40]	2.93	[1.62–5.29]
>20 cigarettes/day	1.43	[0.77–2.65]	2.39	[0.85–6.77]
		P_trend _< 0.001		P_trend _= <0.001
				
Total score for exercise with or without sweating				
Quartile1 (score 2–4)		Reference		Reference
Quartile 2 (score 5)	1.02	[0.72–1.45]	0.64	[0.33–1.25]
Quartile 3 (score 6)	0.96	[0.70–1.33]	0.82	[0.47–1.43]
Quartile 4 (score 7–12)	1.19	[0.89–1.60]	0.67	[0.39–1.16]
		P_trend _= 0.24		P_trend _= 0.23
				
Servings of vegetables, fruit and berries per day				
Quartile 1 (0–1.07 servings per day)		Reference		Reference
Quartile 2 (1.08–1.71 servings per day)	1.14	[0.84–1.54]	0.96	[0.56–1.64]
Quartile 3 (1.72–2.50 servings per day)	1.15	[0.85–1.57]	0.69	[0.37–1.26]
Quartile 4 (At least 2.60 servings per day)	0.90	[0.64–1.27]	0.69	[0.37–1.30]
		P_trend _= 0.63		P_trend _= 0.16
				
Consumption of boiled potatoes				
<1 time/week		Reference		Reference
1–3 times/week	1.15	[0.67–1.98]	0.70	[0.31–1.56]
≥4 times/week	1.38	[0.81–2.34]	0.74	[0.34–1.62]
		P_trend _= 0.09		P_trend _= 0.71
				
Consumption of meat other than poultry				
<1 time/week		Reference		Reference
1–3 times/week	1.07	[0.76–1.50]	0.82	[0.45–1.48]
≥4 times/week	1.24	[0.83–1.87]	0.62	[0.28–1.39]
		P_trend _= 0.26		P_trend _= 0.24

^a^Adjusted for gender, age, bmi, smoking habits, total score for exercise, total consumption of vegetables, fruit and berries, boiled potatoes, poultry, other meat than poultry and fatty fish.
