# Supplementary material for: Lifestyle as a predictor for colonic neoplasia in asymptomatic individuals
Source: BMC Gastroenterol. 2006 Jan 13;6:5. doi: 10.1186/1471-230X-6-5 (PMC1374667; doi:10.1186/1471-230X-6-5)
Supplement: Additional File 1 — The additional file is a word file (name Appendix), in which all questions and reply options, used in this study, are summarised. [file 1471-230X-6-5-S1.doc]

# Appendix

Questions, reply options and encoding used in the study.

| Questions |  | Reply options | Encoding |
| --- | --- | --- | --- |
|  |  |  |  |
| Height in cm |  |  | kg/m^2^ |
| Weight in kg |  |  |  |
| How many hours per week are you in paid work employment? |  |  |  |
| Do you smoke?^1^ |  | [Never smoked]  [Past smoker] [Occasionally]  [Yes, daily] | [1]  [2]  [3] |
| If you are a current smoker, how many cigarettes do smoke per day?^1^ |  | [1-5/day]  [6-10/day]  [11-20/day]  [>20/day] | [4]  [5]  [6] |
| Past smoker, but quit smoking ………. years ago. |  |  |  |
| Do you smoke pipe or cigar/cigarillos daily?^2^ |  | [Yes]  [No] | [1]  [2] |
| During the last three years have you acquired any chronic diseases that have limited your level of activity? E.g. hip disorder or cardiovascular disease. |  | [Yes]  [No] | [1]  [2] |
| How often do you have physical activity for more than 20 minutes (e.g. walking, cycling, jogging or swimming) ? Put only one mark.^3^ |  | [Never]  [<1 time/week]  [1-2 times/week]  [3-4 times/week]  [5-7 times/week]  [>7 times/week] | [1]  [2]  [3] [A]  [4]  [5]  [6] |
| How often do you have physical activity for more than 20 minutes (e.g. walking, cycling, jogging or swimming)? Put only one mark.^3^ |  | [Never]  [<1 time/week]  [1-2 times/week]  [3-4 times/week]  [5-7 times/week]  [>7 times/week] | [1]  [2]  [3] [B]  [4]  [5]  [6] |
| Total score for exercise |  |  | = [A]+[B] |
| How often do you eat these food items? |  |  |  |
| Fruit and berries^3^  Uncooked vegetables^3^  Boiled vegetables^4^ |  | [ Never]  [1-3 times/month]  [1-3 times/week]  [4-6 times/week]  [1-2 times/day]  [>3 times per day] | 0 servings per day  0.07 servings per day  0.29 servings per day  0.71 servings per day  1.5 servings per day  3 servings per day |
| Boiled potatoes  Poultry  Meat other than poultry  Fatty fish  Egg  Cheese  Chocolate  Chips |  | [ Never]  [1-3 times/month]  [1-3 times/week]  [4-6 times/week]  [1-2 times/day]  [>3 times per day] | [1]  [2]  [3] |
| What is your daily intake of the following food items?  Milk (3.9% fat)  Milk light (1.5% fat)  Skimmed milk (0.1% fat)  Slices of bread |  | [Don't drink/eat it]  [<than1/day]  [1-2/day]  [3-4/day]  [5-6/ day]  [7-8/ day]  [>9/ day] | [1]  [2]  [3]  [4]  [5]  [6]  [7] |
| What type of fat do you usually use 1) On your bread and 2) In cooking |  | [Dairy butter]  [Hard margarine]  [Soft/light margarine]  [Oil]  [Don't use fat] | [1]  [2]  [3]  [4]  [5] |
| If you use butter, how many slices of bread do a small package of butter last for (12 grams)? |  | ….Slices of bread |  |
| Have you changed your dietary habits during the last year? If yes, please specify |  | [Yes]  [No] | [1]  [2] |

^1^ The answers from these two questions were merged into one variable with six different categories: [Never smoker]=1, [Past smoker]=2, [Occasional smoker]=3, [Daily smoker consuming 1-10 cigarettes/day]=4, [Daily smoker consuming 11-20 cigarettes/day] =5, [Daily smokers consuming>20 cigarettes/day]=6. This new variable was used in all the analyses. The second question related to current smoking was a follow-up question for the daily smokers. This was emphasised in the questionnaire. Still, almost 31% (n=88) of those stating to be occasional smokers also marked a daily consumption of cigarettes. We chose to classify them as daily smokers consuming 1-10 cigarettes per day (n=86), 11-20 cigarettes per day (n=1), or more than 20 cigarettes per day (n=1). Among ‘past smokers’ a current cigarette consumption level was filled in by 2% (n=17), and all of them were coded as past smokers. Daily smokers, who had failed to mark their daily consumption level, were put in the ‘1-10 cigarettes per day’ category (n=49).

^2^ All pipe smokers (n=36) were current (n=34) or past (n=2) cigarette smokers. Among cigar or cigarillo smokers (n=62), 87% also stated to be daily cigarette consumers.

^3^ These answers were added up to a new variable to give each participant a total score for physical exercise. E.g.: If person NN answered [Never] to the question on: physical exercise without sweating and [1-2 times/week] to the question on: physical exercise with sweating, the total score for exercise would be: 1+3=4 (min-max 2-12).

^4^ The replies on fruits, berries and boiled or uncooked vegetables were recoded to create a mean number of fruits and vegetable servings per day. The reply ‘never’ was recoded to 0 servings/day, ‘1-3 times/month’ was recoded to 0.07 servings/day, ‘1-3 times/week’ was recoded to 0.29 servings/day, 4-6 times/week was recoded to 0.71 servings/day, 1-2 times/day was recoded to 1.5 servings/day and finally >3 times/day was recoded to 3 servings/day. These three answers were added up to a new variable showing the daily average intake of fruits and vegetables.
